# Chitosan as a Natural Preservative in the Food Industry: Advances in Its Technofunctional Properties and Applications

**DOI:** 10.1155/bmri/1232099

**Published:** 2026-07-15

**Authors:** Asik Ikbal, Supratim Chowdhury, Ankures Bhattacharya, Siddhnath Kumar, Vijay Kumar Reddy Surasani, Saranya Vinayagam, Selvamani Palanisamy, Lalitha Gnanasekaran, Sowjanya Muthyam, Pavan Kumar Dara, Hitesh Chopra, Tabarak Malik

**Affiliations:** ^1^ Department of Fish Processing Technology, West Bengal University of Animal & Fishery Sciences, Kolkata, West Bengal, 700037, India, wbuafscl.ac.in; ^2^ Department of Fish Processing Technology, School of Fisheries, Centurion University of Technology and Management, Paralakhemundi, Odisha, India, cutm.ac.in; ^3^ Department of Fish Processing Technology, College of Fisheries, Guru Angad Dev Veterinary and Animal Sciences University, Ludhiana, Punjab, India, gadvasu.in; ^4^ Department of Biosciences, Saveetha School of Engineering, Saveetha Institute of Medical and Technical Sciences, Thandalam, Chennai, India, saveetha.com; ^5^ Department of Pharmaceutical Technology & Centre for Excellence in Nanobio Translational Research, University College of Engineering, Anna University, BIT Campus, Tiruchirappalli, Tamil Nadu, India, annauniv.edu; ^6^ International Center of Nanotechnology and Functional Materials, Instituto de Alta Investigación, Universidad de Tarapacá, Arica, Chile, uta.cl; ^7^ Department of Marine Biology, Vikrama Simhapuri University, Nellore, Andhra Pradesh, India, simhapuriuniv.ac.in; ^8^ Department of Poultry and Food Science, College of Agriculture, Auburn University, Auburn, USA, auburn.edu; ^9^ Center for Research Impact & Outcome, Chitkara College of Pharmacy, Chitkara University, Rajpura, Punjab, India, chitkara.edu.in; ^10^ Department of Biomedical Sciences, Institute of Health, Jimma University, Jimma, Ethiopia, ju.edu.et; ^11^ Division of Research and Development, Lovely Professional University, Phagwara, Punjab, India, lpu.in; ^12^ Department of Microbiology, Graphic Era (Deemed to be University), Dehradun, Uttarakhand, 248002, India, geu.ac.in

**Keywords:** antimicrobial activity, antioxidant activity, biopolymer, food products, fortification, quality

## Abstract

This manuscript investigates the utilization of chitosan (CS) as a preservative in the food industry, covering its sources, extraction methods, structural properties, and preparation techniques. CS, derived from shell wastes of crustaceans and various fungi, offers promising antimicrobial and antioxidant properties due to factors like degree of deacetylation and molecular weight. Its application as a preservative spans across diverse food sectors, showcasing its effectiveness in enhancing food quality and extending shelf life while minimizing reliance on chemical additives. CS fortification demonstrates notable impacts on food composition, including moisture retention, reduced lipid oxidation, and improved protein functionality. Notably, its application in the meat and seafood industries proves effective in inhibiting bacterial growth and preserving product freshness. Additionally, the antioxidant activity of CS, influenced by its structural characteristics and supplementation with natural compounds, contributes to its role as a secondary antioxidant in food products. By summarizing existing studies and research, this document provides a comprehensive understanding of CS′s multifaceted applications and benefits in enhancing food quality and promoting sustainable practices in food preservation.

## 1. Introduction

The escalating global population, projected to reach 9.7 billion by 2050, coupled with increasing consumer awareness regarding food safety and environmental sustainability, has intensified the search for natural alternatives to synthetic food preservatives. Traditional chemical preservatives, although effective in extending shelf life, have raised concerns regarding potential health risks, allergenicity, and environmental impact. This paradigm shift has directed scientific attention toward biopolymer‐based preservation systems that can maintain food quality while addressing sustainability imperatives. Among various biopolymers investigated, chitosan (CS)—a linear polysaccharide derived from the deacetylation of chitin found abundantly in crustacean shells and fungal cell walls—has emerged as a particularly promising candidate for food preservation applications [1, 2]. The technofunctional properties of CS are intrinsically linked to its molecular architecture, particularly its degree of deacetylation (DD), which typically ranges from 70% to 95%, and molecular weight (Mw), which can vary from low (< 50 kDa) to high (> 300 kDa) depending on the source and extraction method employed. These structural parameters critically determine CS′s solubility profile, viscosity, charge density, and consequently, its antimicrobial efficacy and film‐forming capacity. The presence of free amino groups (–NH_2_) along the polymer backbone, resulting from deacetylation, confers CS with cationic properties at acidic pH (pKa ≈ 6.5), enabling electrostatic interactions with negatively charged microbial cell membranes and anionic food components. These structure‐function relationships directly influence CS′s antimicrobial and antioxidant properties, which are crucial for its role in food preservation [3]. The presence of free amino groups (–NH_2_) along the polymer backbone, resulting from deacetylation, confers CS with cationic properties at acidic pH (pKa ≈ 6.5), enabling electrostatic interactions with negatively charged microbial cell membranes and anionic food components. These structure‐function relationships directly influence CS′s antimicrobial and antioxidant properties, which are crucial for its role in food preservation [4]. The ability of CS to form semipermeable films and edible coatings through inter‐ and intramolecular hydrogen bonding, combined with its excellent oxygen barrier properties, enables the creation of protective matrices that restrict microbial infiltration, minimize moisture migration, and retard oxidative deterioration. This multifunctional character underpins CS′s application in extending the shelf life of diverse food products, ranging from fresh produce to processed meat and seafood items [5, 6].

**Figure 1:**
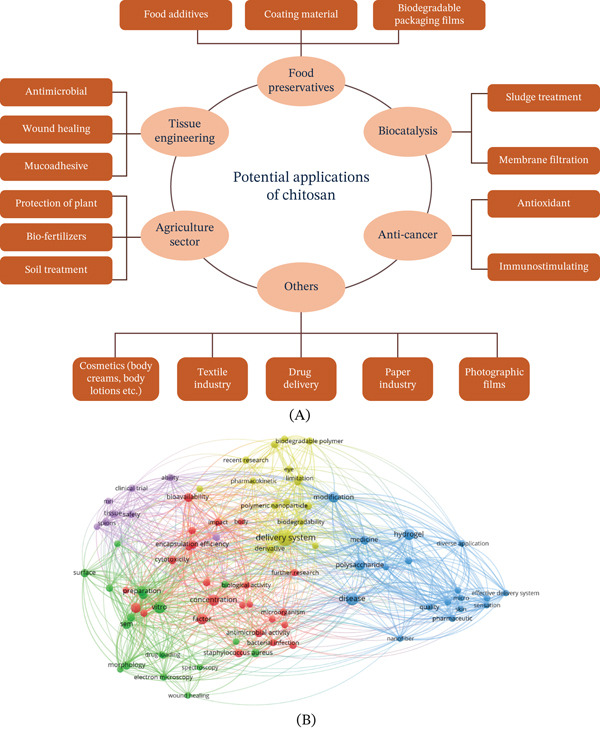
(A) Applications of chitosan in different sectors. The diagram illustrates the versatility of chitosan across various industries, including its use as a natural preservative in food, a flocculant in wastewater treatment, a biostimulant in agriculture, and a biocompatible scaffold in biomedical engineering. (B) Chitosan: Research and therapeutic applications (VOS viewer 04.02.2026). A bibliometric network visualisation highlighting the major clusters of research focus. The nodes represent key terms such as drug delivery, antimicrobial activity, and tissue engineering, showing the multidisciplinary scope of chitosan research.

The antimicrobial activity (A_M_A_C_) of CS represents one of the most extensively investigated aspects of its preservative functionality. The prevailing mechanistic hypothesis attributes CS′s bactericidal and bacteriostatic effects to the electrostatic interaction between protonated amino groups and negatively charged phospholipid components of microbial cell membranes, leading to membrane disruption, increased permeability, and subsequent leakage of intracellular constituents. This mechanism shows broad‐spectrum activity against both Gram‐positive and Gram‐negative bacteria, including prevalent foodborne pathogens such as *Escherichia coli*, *Listeria monocytogenes*, *Salmonella* spp., and *Staphylococcus aureus* [7] rendering CS particularly valuable for preserving highly perishable products such as seafood, meat, and dairy items. Complementing its antimicrobial properties, CS exhibits significant antioxidant activity (A_X_A_C_) through multiple mechanisms, including radical scavenging (via donation of hydrogen atoms from hydroxyl and amino groups), metal chelation (complexation with prooxidant transition metals such as Fe^2+^ and Cu^2+^), and direct interaction with lipid radicals. These antioxidant mechanisms are crucial for mitigating lipid oxidation (LIP_O_), one of the primary deteriorative pathways in lipid‐containing foods, thereby preserving nutritional quality, preventing off‐flavor development, and extending shelf life [8]. Beyond its direct antimicrobial and antioxidant functions, CS incorporation demonstrably influences the proximate composition and physicochemical attributes of fortified food products. CS‐based treatments can modulate moisture content (MC) through their hygroscopic nature and film‐forming properties, alter lipid profiles by reducing oxidative degradation, and interact with protein matrices through electrostatic and hydrogen bonding interactions, potentially affecting protein denaturation, aggregation, and functional properties [9]. The efficacy of CS‐based preservation strategies can be systematically evaluated through comprehensive monitoring of critical quality indicators, including pH dynamics, peroxide value (PV) as an index of primary LIP_O_, total volatile base nitrogen (TVBN) reflecting protein degradation and microbial activity, thiobarbituric acid‐reactive species (TBARS) quantifying secondary LIP_O_ products, and total plate count (TPC) assessing microbiological quality. These parameters collectively provide a multidimensional assessment of CS′s preservative functionality. Additionally, CS treatment can significantly influence cooking characteristics, color stability (through inhibition of enzymatic and nonenzymatic browning), textural properties (via modification of water‐holding capacity and protein–polysaccharide interactions), and overall sensory profiles, which are critical determinants of consumer acceptance and commercial viability [10]. Importantly, the inherent biocompatibility and biodegradability of CS align with contemporary sustainability imperatives in food packaging and preservation, positioning it as an environmentally benign alternative to petroleum‐derived synthetic preservatives and packaging materials. The complete degradation of CS through enzymatic and microbial pathways minimizes environmental accumulation and supports circular economy principles (Figure 1a), addressing regulatory and consumer demands for sustainable food production practices [11].

Despite the substantial body of research demonstrating CS′s potential in food preservation, several critical knowledge gaps persist that limit its widespread industrial adoption, such as (a) the considerable variability in reported antimicrobial and antioxidant efficacies, attributable to inconsistencies in CS source materials, extraction protocols, and structural characterization (particularly DD and Mw determination); (b) though numerous studies have examined CS applications in specific food matrices, systematic comparative analyses elucidating the relationship between CS structural parameters and performance across different food systems remain limited; (c) the pH‐dependent solubility of CS (soluble only under acidic conditions) presents a significant technological challenge for applications in neutral or alkaline food products, necessitating chemical modification or nanoencapsulation strategies that require comprehensive evaluation; (d) emerging applications of CS as a carrier matrix for codelivery of multiple bioactive compounds (e.g., essential oils, plant extracts, antimicrobial peptides) to achieve synergistic preservation effects remain incompletely characterized in terms of stability, release kinetics, and food matrix interactions; and finally, (e) the translation of laboratory‐scale findings to industrial‐scale applications requires addressing scalability, cost‐effectiveness, regulatory compliance, and consumer acceptance, aspects that warrant integrated technoeconomic and sensory evaluation.

In view of these knowledge gaps, this comprehensive review aims to (i) systematically evaluate the current understanding of CS sources, extraction methodologies, and structure‐property relationships governing its technofunctional attributes; (ii) critically analyze the mechanisms underlying CS′s antimicrobial and antioxidant activities in food systems; (iii) examine the effects of CS incorporation on proximate composition, physicochemical parameters, and sensory characteristics across diverse food categories including meat, seafood, dairy, fruits, vegetables, and processed foods; (iv) assess emerging trends in CS derivative development, nanoformulations, and composite systems designed to enhance functionality and overcome solubility limitations; and (v) identify research priorities and technological innovations necessary to facilitate industrial‐scale implementation of CS‐based preservation technologies. By synthesizing current knowledge while highlighting critical research gaps and future directions (Figure 1b), this review provides a foundational resource for researchers, food technologists, and policymakers engaged in advancing sustainable, natural preservation strategies for the food industry.

## 2. An Overview of CS

### 2.1. Sources of CS

**Figure 2:**
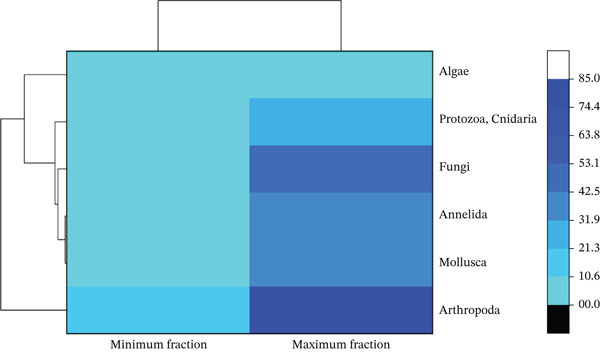
Chitosan abundance in different sources using a dendrogram. The chart categorizes the primary biological sources of chitin/chitosan, distinguishing between marine sources and terrestrial/microbial sources, which vary in availability and extraction yield.

Chitin [poly‐*β*‐(1 → 4)‐N‐acetyl‐D‐glucosamine] is one of the most abundant natural biopolymers. It is found naturally in eukaryotes, basically in a wide range of animals, including ciliates, amoebae, chrysophytes, certain algae, yeasts, worms, insects, and molluscs (Figure 2) [2, 12]. It is not found in vertebrates, plants, or prokaryotes. Chitin is the precursor or major source of CS, which is gained by removing the acetyl group (CH_3_‐CO) from chitin. CS is more abundant in fungi such as zygomycetes and mucorales, e.g., *Absidiacoerulae*, *Gongronellabutleri*, *Mucor rouxii*, *Aspergillus niger*, and *Rhizopus* sp KNO_1_ and KNO_2_. The maximum extractable CS was found in *A. niger* during the late exponential phase [13]. Recently, on a commercial scale, CS has been made by chitin deacetylation from fungal origin and is still an innovative technology.

**Figure 3:**
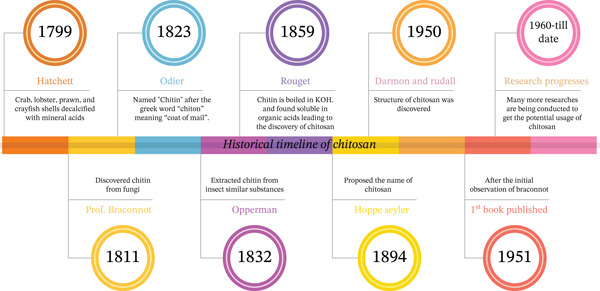
Historical timeline of chitosan discovery and development.

Shell wastes of giant tiger prawn (*Penaeus monodon*) and Indian prawn (*Fenneropenaeus indicus*) were observed to be used for CS extraction [14, 15]. Several studies indicated that processing waste of shrimps, crabs, lobsters, crayfish, and oysters has been utilized for the extraction of CS [16]. Chitin content depends on the type of species; crustacean shell waste contains 30%–50% CaCO_3_ and 20%–30% chitin by weight. It is reported that *Procambarusclarkii* contains 20%–23% of chitin, whereas lobster contains 60%–75% [17]. The historical timeline of CS is given in Figure 3.

### 2.2. Structure and Derivatives of CS

**Figure 4:**
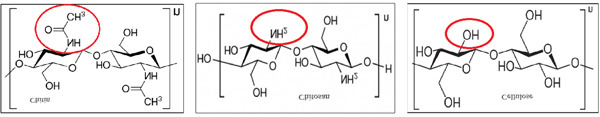
Structure of chitin, chitosan, and cellulose. A chemical comparison showing the structural similarities between the three biopolymers. The primary difference lies at the C‐2 position: cellulose contains a hydroxyl group (−OH−OH), chitin contains an acetamido group (−NHCOCH3−NHCOCH3), and chitosan contains a free amino group (−NH2−NH2), resulting from deacetylation.

After deacetylation, the repeating units in the chitin molecule are primarily without the acetyl functional group, as −1,4‐D‐glucosamine (Figure 4). The degree of acetylation (DA) is well‐defined as the mole fraction of N‐acetylated repeating units in polysaccharides, whereas DD is defined as the percentage of repeating units of 1,4‐D‐glucosamine in polysaccharides. [18]. Consequently, the DD is inversely related to the DA; a fully deacetylated CS would have a DD of 100% and a DA of 0%. Commercially available CSs have DD between 70%–90% [19]. DD is an important characteristic, as several studies have found that CS with a greater DD has better biological effects and is more water‐soluble [20]. CS with greater DD suggests a higher amino group concentration and protonation of the –NH_2_ functional group, and these are required for CS′s biological effects and water solubility (WS). Aside from DD, Mw is another essential factor that determines CS bioactivity. Like DA, low Mw CS (LMCS) usually has stronger bioactivities than higher Mw CS (HMCS) [21, 22]. Mw of CS is inversely proportional to the solubility of the carbohydrate molecule [23]. CS with a Mw < 30 kDa is water soluble in its free amine form without acidification. However, even in the sub‐30 kDa range, CS with Mw > 22 kDa only has limited WS, whereas CS with Mw < 9 kDa has much‐improved WS. If Mw of CS > 30 kDa, protonation of the amino group by an acid is required to dissolve it in water. Apart from phosphoric acid, acetic acid is the most widely utilized acid for dissolving [24, 25].

CS, one of the most significant chitin derivatives, is a polycationic biopolymer produced by partial or total deacetylation of chitin in an alkaline. The presence of the acetyl amine (–NHCOCH_3_) rather than the hydroxyl (–OH) in the cellulose structure is the only distinction between cellulose and chitin biopolymer [26] (Figure 4). In addition to this benefit, CS′s application is highlighted by the fact that its chain structure contains both –OH and –NH_2_ groups and these groups may be changed in a variety of ways [16]. CS is a natural, secure, and affordable raw biopolymer utilized in various industrial sectors, including food, medicines, cosmetics, agriculture, wastewater treatment, and textiles. In addition to possessing antiviral, antibacterial, and antifungal qualities, CS is a powerful tool for disease prevention and management since it strengthens plants′ natural defenses. Additionally, CS is being utilized to enhance agriculture due to its ability to chelate metal ions in the environment.

### 2.3. Properties of CS

CS is a biocompatible and biodegradable biopolymer that degrades progressively to nontoxic byproducts. Processing conditions and several factors, such as Mw, DD, crystallinity, ionization/free amino group, define the physico‐chemical and morphological properties of CS [1]. The Mw and DD of CS were in the range of 50–2000 kDa and 40%–98% [27]. DD is one of the important properties that has a linear relationship with its molecular properties. DD influences the flexible nature and electrostatic interactions, which directly tend to change the mechanical properties of CS. Conversely, CS with a lower DD retains a higher number of acetamido groups; these groups promote extensive hydrophobic interactions and intermolecular hydrogen bonding, which lead to increased chain entanglement and reduced solubility. Higher temperature affects the viscosity and shear rates of CS [28]. The viscosity of commercial CS [1% (w/v) in 1% acetic acid at 25°C] is in the range of 10–1000 mPa.s [29]. CS dissolves in mild acids and becomes insoluble at alkaline conditions. Several factors, such as pKa value and solvent strength, govern the solubility of CS [30].

CS is defined as chitin that has been sufficiently N‐deacetylated to make it soluble in diluted aqueous acids. Chitin and CS vary primarily in their solubility. Insoluble in water, alkaline media, and even organic solvents is pure, native CS (pKa about 6.3). However, by neutralizing CS with organic acids or inorganic acids like hydrochloric acid, water‐soluble salts of CS may be created. The pH‐dependent solubility of CS is due to its amino groups (–NH_2_), which, when dissolved at pH 6 or lower, protonate to form cationic amine groups (–NH^3+^), increase intermolecular electric repulsion and produce polycationic soluble polysaccharide [2, 31].

### 2.4. Production of CS

**Figure 5:**
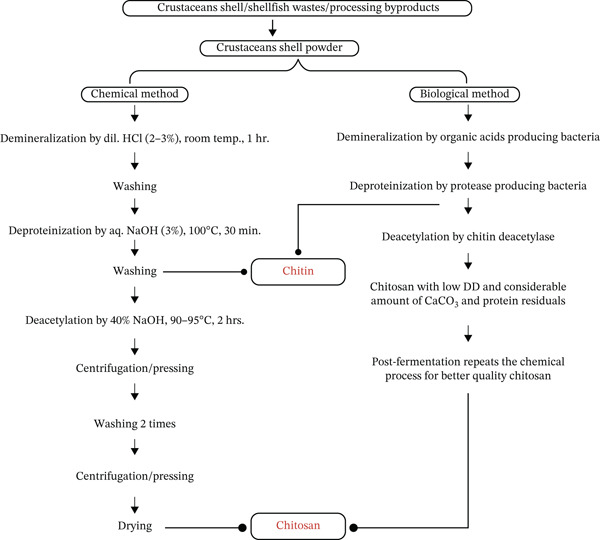
Production process of chitosan.

The synthesis of CS or chitin from marine processing waste has been widely studied. In general, chemical and biological approaches are used to extract CS from crustacea byproducts (Figure 5).

#### 2.4.1. Chemical Processes

The chemical extraction of CS involves three primary steps: demineralization, deproteinization, and deacetylation. Demineralization entails the removal of calcium carbonate using dilute acids (e.g., 10% HCl) at ambient temperature. This is followed by deproteinization, where proteins are removed using dilute alkaline solutions (e.g., 1%–10% NaOH) at elevated temperatures (65°C–100°C). Finally, deacetylation converts chitin into CS by hydrolyzing the acetamido groups. This is achieved using concentrated alkali (40%–50% NaOH) at high temperatures (100°C or higher) for extended periods. This step removes the acetyl group, exposing the amino group responsible for CS′s polycationic nature and biological activity. The specific reaction conditions (time, temperature, alkali concentration) are critical, as they directly determine the Mwand DD of the final product [32, 33].

#### 2.4.2. Biological Processes

In addition to chemical approaches, biological methods (e.g., enzymatic and fermentation processes) can be used to make CS from crustacean wastes. Enzymatic procedures use the same demineralization mechanism as chemical approaches, namely, the use of acid to remove CaCO_3_ from the shell. Nonetheless, this method contains the application of enzymes for deproteinization and deacetylation reactions at a low temperature (25°C–59°C) [34–36]. Proteases play an important role in chitin extraction process. Plant, microbial, and animal sources are the most common sources of proteolytic enzymes, which alter the reactant′s accessibility. In the deproteinization stage, both pure and crude proteases are utilized. Commercially, enzymes are more expensive than crude proteolytic enzymes, which are cheaper as well as more efficient due to coexisting proteases. Most of the crude proteases come from microbial sources and fish processing waste viscera. It is noteworthy to emphasize that enzymatic methods are less efficient than chemical approaches, with roughly 5%–10% residual protein often remaining with the recovered chitin. Deproteinization through fermentation can be performed by endogenous microorganisms or by adding specific strains of microbes which can reduce the cost of utilizing enzymes. Depending on the microbial strains utilized, fermentation methods can be lactic acid and nonlactic acid fermentation methods. Lactic acid fermentation procedures have been observed to take anywhere from a few days to a few weeks [37].

Chemical approaches provide the advantages of shorter process durations, easier manufacturing procedures, and CS can be produced with medium to lower Mw and greater DD. The disadvantages of chemical procedures, on the other hand, are that the reaction process generally involves toxic or corrosive chemicals (e.g., HCl and NaOH), and as a result, the byproducts of the reaction are toxic or corrosive.

## 3. CS as a Game‐Changer in Food Preservation

In the relentless pursuit of safer and higher‐quality food products, CS stands out as a beacon of innovation in the global food industry. Its remarkable versatility has propelled it into the spotlight, with nations like Japan, Korea, and Italy embracing its manifold benefits as a food additive. Today′s consumers demand nothing short of excellence in their food choices, igniting a fervent quest within the food industry to elevate shelf life and quality standards [38].

The applications of CS in the food sector are as diverse as they are impactful. With a paramount emphasis on reducing or eliminating chemical additives, CS emerges as a natural ally, meeting the escalating demand for cleaner food options. Beyond its role as a mere ingredient, CS dons the mantle of a secondary antioxidant, intervening in the early stages of LIP_O_ and preserving the pristine quality of food products. CS′s modus operandi, disrupting cell permeability through a delicate dance of positive and negative charges, unveils a cascade of benefits. Intracellular components are liberated, whereas the synthesis of vital cellular components grinds to a halt. Moreover, CS′s affinity for metals and essential nutrients amplifies its preservative prowess, safeguarding food integrity on multiple fronts. In a landscape brimming with innovation, CS assumes various forms and functions. Whether dissolved in acetic acid to create potent solutions or woven into the very fabric of food packaging materials, its versatility knows no bounds [39, 40]. The advent of CS nano/microparticles, orchestrated through intricate ionic cross‐linking, adds yet another dimension to its arsenal [41]. Although these innovations hold promise, the timeless efficacy of CS solutions remains unchallenged, standing as a testament to its enduring relevance. The battleground against microbial foes sees CS emerge as a formidable adversary, particularly excelling against Gram‐negative bacteria. Studies have showcased its prowess in extending the shelf life of delicate marine products like oysters, mitigating the proliferation of common cold storage microorganisms such as *Pseudomonas* and *Shewanella* [42]. This is not merely a triumph of science but a testament to CS′s tangible impact on food safety and sustainability.

Yet, amidst the triumphs, caution is warranted. The LD_50_ of CS, although reassuringly high in mice, prompts prudence in human consumption. Clinical trials offer reassurance, indicating the safety of moderate CS intake. However, rigorous oversight remains imperative as we navigate the evolving landscape of food preservation. In the grand tapestry of food science, CS emerges not just as a mere ingredient but as a catalyst for change. Its multifaceted role in enhancing food safety, quality, and sustainability underscores its indispensability in the modern food industry. As we march forward, let CS serve as a beacon of inspiration, illuminating the path toward a future where food is not just sustenance but a celebration of nature′s bounty, preserved and protected by the marvels of science. In the following sections, the application of CS as a preservative in different food categories has been discussed.

### 3.1. Meat Industry

Meat and its processed derivatives stand as vulnerable targets for LIP_O_, a process that hastens the emergence of rancid or “warmed‐over” flavors. Extensive research has delved into the intricate interplay between CS and meat, unveiling its capacity to counteract these deleterious effects. Revered for its A_X_A_C_, CS has showcased its ability to impede LIP_O_ and suppress the proliferation of spoilage bacteria during meat storage. Multiple studies have underscored the significant enhancements in meat′s storage robustness through CS. For instance, Darmadji and Izumimoto [43] observed a notable reduction, nearly 70%, in TBA (thiobarbituric acid) value, a pivotal marker of LIP_O_, following the addition of 1.0% CS to beef samples stored for 3 days at 40°C. Impressively, beef samples supplemented with CS maintained stable TBA values even after 10 days of storage. Additionally, CS exerted a positive influence on the red color development of beef during storage. CS exerted a positive influence on the red color development of beef during storage. This is attributed to the ability of CS to chelate iron ions released from heme pigments, thereby inhibiting the oxidation of myoglobin to metamyoglobin, which is responsible for the brown discoloration.

The introduction of CS into meat processing has yielded commendable strides in microbial inhibition and preservation. Youn et al. [44] showcased that integrating 1% CS into spicy beef formulations prolonged their shelf life by decreasing microbial counts and mitigating LIP_O_ at 40°C for 10 days. Similarly, Sagoo et al. [45] substantiated CS′s efficacy as a microbial growth inhibitor in chilled comminuted pork products, with its impact contingent upon concentration. Noteworthy findings emerged as the addition of CS glutamate to minced pork mixtures led to substantial reductions in total viable counts, yeasts and molds, and lactic acid bacteria, thereby fortifying the microbial stability of the product. Moreover, CS supplementation has proven effective in mitigating the risk of *Clostridium perfringens* spore germination and outgrowth in ground beef or turkey under conditions of abusive cooling. Juneja et al. [46] reported that adding 3% CS glutamate into meat curtailed the temperature danger zone from 54.4°C to 7.2°C within 12, 15, or 18 h, thus bolstering food safety measures. Similarly, Sagoo et al. [45] evaluated the storage stability of pork treated with CS solution. Pork pieces were immersed for 1.0 min in CS (Mw: 5, 30, 120 kDa) solutions (0.1%, 0.5%, 1.0%), followed by storage at 10°C for 8 days. The findings revealed that immersing pork in 1.0% solutions of 30 and 120 kDa CS significantly extended its shelf life and conferred A_X_A_C_.

Exploring further applications, several researchers investigated the use of CS as a curing agent in sausage production [47–49]. Their studies suggested that CS could potentially reduce or replace the need for sodium nitrite without compromising the preservative effect or color development of sausages. For instance, Youn [47] demonstrated that incorporating 0.2% CS (30 kDa, DD = 92*%*) dissolved in 0.3% lactic acid reduced sodium nitrite levels by half without compromising sausage quality or storage stability. Additionally, CS supplementation significantly reduced residual nitrite levels in sausages, thereby mitigating the risk of nitrosamine formation, a potent toxin harmful to human health [49]. The preservation efficacy of CS in sausage production appears to be influenced by its Mw. According to Youn et al. [50] CS′s preservation activity increased with higher Mw (1, 5, 30, and 120 kDa), with no observed preservation effect for CS (Mw: 1 kDa). Lin and Chao [51] found that adding CS with various Mw (150, 600, 1250 kDa) to Chinese‐style sausages had no discernible effect on textural and sensory characteristics, indicating its potential as a versatile additive. Similarly, Jo et al. [52] reported no significant differences in sensory attributes between sausages with and without water‐soluble CS oligomer (5 kDa, 0.2%). However, sausages containing CS oligomer exhibited reduced LIP_O_ during storage compared to control sausages. Furthermore, CS has demonstrated A_X_A_C_ in emulsion‐type sausages. Youn et al. [50] observed that A_X_A_C_ of CS increased with higher Mw (1, 5, 30, 120 kDa) and concentrations (0.2%, 0.35%, and 0.5%), highlighting its potential to enhance the oxidative stability of emulsified sausages.

In summary, CS emerges as a promising natural solution for elevating the quality and safety of meat products. Its capacity to hinder LIP_O_, inhibit microbial growth, and augment preservation underscores its potential as an asset in the meat industry′s arsenal. As ongoing research in this sphere evolves, the widespread adoption of CS‐based interventions holds the promise of revolutionizing meat preservation practices while upholding consumer satisfaction and safety.

### 3.2. Seafood Products

Seafood products are extremely prone to quality deterioration due to the presence of hematin compounds and metal ions in the fish muscle, which catalyze the oxidation of unsaturated fatty acids. This susceptibility to LIP_O_, combined with factors such as autolysis, microbial contamination, and protein functionality loss, poses significant challenges to maintaining seafood quality. Kamil et al. [53] examined A_X_A_C_ of CSs with varying viscosities (360, 57, and 14 cP; corresponding to Mw: 1800, 960, and 660 kDa) in cooked herring flesh. The addition of CS (50, 100, and 200 ppm) to fish flesh was found to effectively inhibit LIP_O_ during storage at 40°C, with the 14 cP CS demonstrating the highest efficacy. Notably, A_X_A_C_ of CS was dependent on both its Mw and concentration. Similarly, Kim and Thomas [54] investigated A_X_A_C_ of CS in salmon, observing variations based on Mw (30, 90, and 120 kDa) and concentration (0.2%, 0.5%, and 1.0%). CS was found to mitigate LIP_O_ by chelating ferrous ions and inhibiting their prooxidant activity. A_X_A_C_ of CS improved at higher concentrations, with 30 kDa CS exhibiting the highest efficacy. Further research by Jeon et al. [55] explored the effect of CS coatings on the shelf life of fresh fillets of Atlantic cod and herring. CS coatings (360 and 57 cP) significantly reduced LIP_O_, chemical deterioration, and microbial growth compared to uncoated samples. Additionally, Tsai et al. [56] and Sathivel et al. [57] demonstrated that CS coatings extended the shelf life of salmon fillets by reducing moisture loss and delaying LIP_O_ during storage and freezing. López‐Caballero et al. [58] found that coating fish patties with a CS–gelatin combination slowed down spoilage, highlighting the potential of CS‐based coatings to preserve seafood quality.

In conclusion, CS emerges as a promising preservative agent for seafood products, offering A_X_A_C_ that can effectively mitigate LIP_O_ and extend shelf life. Its application in edible coatings shows great potential for enhancing the quality and safety of seafood during storage and transportation.

### 3.3. Cereal‐Based Products

#### 3.3.1. Bread

Bread shelf life is often restricted due to microbial development and staling [59]. Staling is a phrase used to indicate the loss of flavor quality and the bread′s texture over time. Bread staleness is a complicated phenomenon in the food industry [60]. There have been reports of CS being used to extend shelf life of bread by preventing starch retrogradation and/or suppressing microbial development. Park et al. [61] looked into the effect of a CS (493 kDa) coating on baguette shelf life. After molding, the dough was coated with a brush containing 0.5%, 1.0%, or 1.5% CS in 1.0% acetic acid. During storage for 36 h at 25°C, baguettes coated with CS, particularly 1% CS, showed less weight loss, hardness, and retrogradation than the control. This is most likely owing to CS′s moisture barrier characteristics [62]. CS coating acts as a barrier to moisture transmission through the bread surface, lowering weight loss while also delaying hardness. Furthermore, the hydroxyl and amino groups of CS can form hydrogen bonds with starch amylose, thereby interfering with the reassociation of starch molecules (retrogradation) and delaying the staling process. When compared to the control (12 h), shelf life of 1% CS‐treated baguette was extended by 24 h (36 h). In a different experiment, Park et al. [61] discovered that a baguette coated with 1% CS oligomer (2 kDa) dissolved in distilled water had a shelf life of 24 h, compared to 12 h for the control.

According to Ahn et al. [63] CS coating extended Shelf life and increased the quality of bread by suppressing microbial growth and delaying autooxidation and retrogradation. After 8 days of storage at room temperature, bread coated with 1% and 2% CS (120 kDa, DD = 85*%*) dissolved in 0.3% lactic acid had lower total bacterial counts and TBARS and higher MC than the control. Mold development was identified in the control after 4 days of storage, but not in the bread coated with 1% and 2% CS for the entire 8‐day period. The Shelf life of bread may be affected by Mw and concentration of CS. Lee et al. [64] examined how different Mw CSs affected Shelf life of wheat bread at varying concentrations. HMCS (30 and 120 kDa) extended Shelf life of bread better than LMCS (1 and 5 kDa).

#### 3.3.2. Noodles

Lee and No [65] investigated the impact of CS (Mw: 37 kDa) on shelf life and quality of wet noodles. CS, dissolved in 1% acetic acid, was added to wheat flour at varying concentrations. During storage at 18°C for 6 days, there was a marginal reduction in MC in wet noodles irrespective of CS concentration. However, an increase in CS content from 0% to 0.70% led to lower viable cells after 6 days of storage at 18°C. Wet noodles with CS rates of 0.17%, 0.35%, 0.52%, and 0.70% exhibited extended shelf life by 1, 2, 3, and 3 days, respectively, compared to the control. Among the samples, wet noodles with 0.35% CS demonstrated the highest sensory quality. Furthermore, Lee et al. [66] demonstrated that wet noodles containing CS (Mw: 37 kDa) dissolved in 1% lactic acid (0.1% or 0.5% concentration) could be preserved for over 80 days, in contrast to only 7 days for the control. These findings underscored the efficacy of CS as an efficient preservative in wet noodles due to its A_M_A_C_.

#### 3.3.3. Rice Cake

In a study by Lee et al. [66], CS treatment significantly enhanced shelf life of white rice cake. Preceding vacuum packing, white rice cakes underwent dipping treatments in 95% alcohol, 1% lactic acid, or 1% and/or 2% CS (Mw: 37 kDa, dissolved in 1% lactic acid) for 10 s. After storage at 4°C for various durations, it was observed that TPC for the control (no treatment), alcohol‐treated, and 1% lactic acid‐treated white rice cakes exceeded the first putrefactive threshold level of 1 × 10^6^ CFU/g. However, TPC of white rice cakes treated with 1% and 2% CS remained below this threshold even after 76 days. Although dipping treatments, regardless of solution type, negatively impacted the sensory acceptance of white rice cakes, A_M_A_C_ of CS was evident. Additionally, Nam and Woo [67] investigated the effect of CS oligosaccharide addition (2%, 4%, and 6%) on the quality of jeung‐pyun, a traditional Korean fermented rice cake leavened by yeast. The addition of 2% CS oligosaccharide positively influenced the quality of jeung‐pyun based on sensory evaluation and rheological parameters.

### 3.4. Antimicrobial Mechanisms of CS

**Figure 6:**
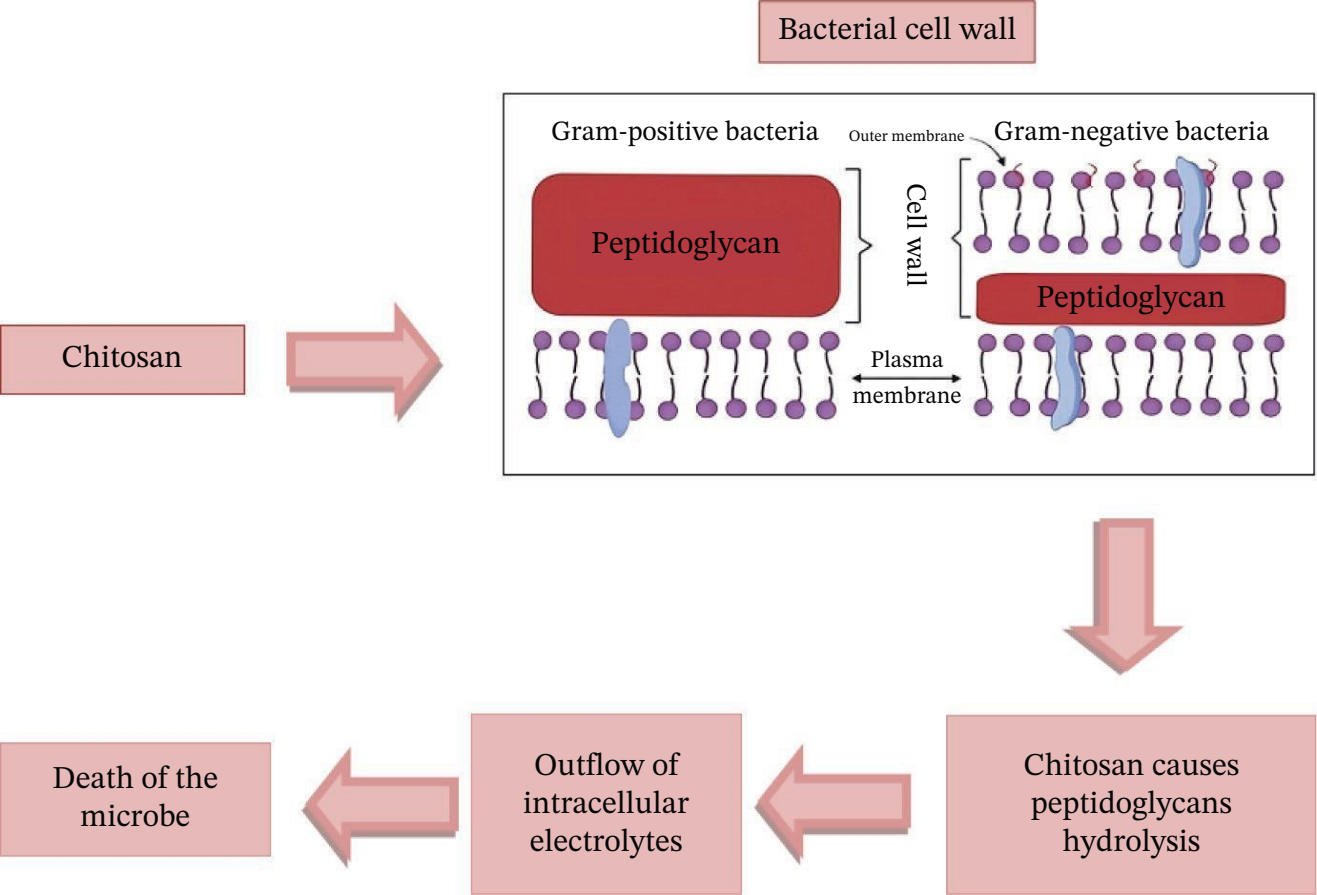
Proposed antimicrobial mechanism of chitosan. The diagram depicts three primary modes of action: (1) electrostatic interaction between positively charged chitosan amine groups and negatively charged microbial cell membranes, causing leakage of intracellular constituents; (2) chelation of essential metal ions and nutrients; and (3) penetration into the cell to bind DNA and inhibit mRNA synthesis.

CS has garnered considerable interest as a potential natural food preservative due to its ability to combat a wide spectrum of foodborne filamentous fungi, yeast, and bacteria [45]. Despite numerous hypotheses, the precise mechanism underlying A_M_A_C_ of CS remains largely unexplored. It is widely suggested that a change in cell permeability may be the primary mechanism (Figure 6). This change occurs as positively charged CS molecules interact with negatively charged microbial cell membranes, resulting in altered permeability. Consequently, this interaction leads to the leakage of intracellular substances such as proteins [68]. Other potential mechanisms include the interaction of CS′s hydrolysis products with microbial DNA, inhibiting mRNA and protein synthesis [69], as well as the chelation of metals, spore elements, and essential nutrients [70]. CS′s efficacy extends to both Gram‐positive and Gram‐negative bacteria, outperforming its effectiveness against fungi in terms of A_M_A_C_ [71].

Recent investigations into A_M_A_C_ of CS have highlighted its superiority over CS oligomers in inhibiting bacterial growth [71]. Furthermore, A_M_A_C_ of CS depends on several factors, including its Mw, DD, pathogen type, pH of the medium, source, and concentration [3]. Higher DD results in a heightened positive charge, facilitating electrostatic binding to membranes and enhancing permeabilization effects. Conversely, HMCS leads to reduced penetration into the cell nucleus [72–75]. Besides, A_M_A_C_ of CS in food and agricultural applications is influenced by various internal and external factors, including microbial species, physiological conditions, pH, temperature, ionic strength, presence of metal ions, ethylenediaminetetraacetic acid (EDTA), organic matter, as well as the solvent and concentration used. A critical factor influencing its A_M_A_C_ is the pH level. CS is a cationic polyelectrolyte; its amino groups (pKapKa ~6.3–6.5) become protonated (NH3 + NH3+ ) in acidic environments. These positively charged groups interact electrostatically with anionic components (lipopolysaccharides, proteins) on the bacterial surface, disrupting membrane integrity. This explains why CS solutions with lower pH demonstrate heightened A_M_A_C_. Regarding sources, crustacean‐derived CS often exhibits superior efficacy compared to insect‐derived CS against pathogens like *Salmonella*, likely due to differences in Mw distribution and the purity of the extraction achieved from the different chitin structures [76]. Moreover, it was observed that CS solutions with lower pH demonstrated heightened A_M_A_C_ compared to those with higher pH [77]. In a broader context, research has explored the response of various pathogenic bacteria to different levels of CS across a range of pH. For instance, *L. monocytogenes*, a pathogenic bacterium, has demonstrated resistance to CS at pH 6.5 but exhibited significant inhibition at pH 5.5. This highlights the pH‐dependent A_M_A_C_ of CS and emphasizes the need for pH optimization to maximize its efficacy against specific bacterial strains [78].

Moreover, the size of the bacterial inoculum has been identified as a potential factor influencing A_M_A_C_ of CS. Studies suggest that higher bacterial loads may diminish the effectiveness of CS, highlighting the importance of considering bacterial density when assessing its A_M_A_C_ [76]. CS exhibits remarkable A_M_A_C_ against both Gram‐positive and Gram‐negative bacteria, including notable species like *Bacillus cereus*, *S. aureus*, *Salmonella typhimurium*, and *E. coli* [79]. Teichoic acid, a vital component of the peptidoglycan layer in Gram‐positive bacteria, plays a crucial role in cell division and structural integrity. Although scientists have started to uncover how CS interacts with teichoic acid, the exact mechanisms are still somewhat unclear. It’s proposed that due to its larger size, CS may form noncovalent bonds with teichoic acid molecules on the bacterial surface, potentially disrupting the cell membrane and leading to cell lysis, particularly evident in Gram‐positive bacteria [80]. Gram‐negative bacteria, however, appear to encounter CS differently. At higher pH, CS might bind to cations, potentially compromising the cell wall′s integrity and nutrient uptake. Additionally, interactions with anions linked to lipopolysaccharides in the outer membrane could induce electrostatic disruptions, further destabilizing the membrane. These combined effects could result in leakage of cellular contents and eventual membrane breakdown, disrupting vital cellular processes [81]. Furthermore, CS′s impact on the cell membrane may increase the permeability of Gram‐negative bacteria, interfering with essential processes like DNA and RNA synthesis and prompting intracellular responses.

Despite the extensive documentation of A_M_A_C_ for CS in lab settings, its performance in real‐world food matrices remains a subject of ongoing investigation. Recent studies, such as the work by Hosseinnejad and Jafari [82] have delved into understanding how various food components, such as starch, protein, oil, and sodium chloride, may influence A_M_A_C_ of CS. Such insights are crucial for optimizing CS′s application as a natural preservative in food systems, paving the way for safer and more sustainable food preservation methods.

**Figure 7:**
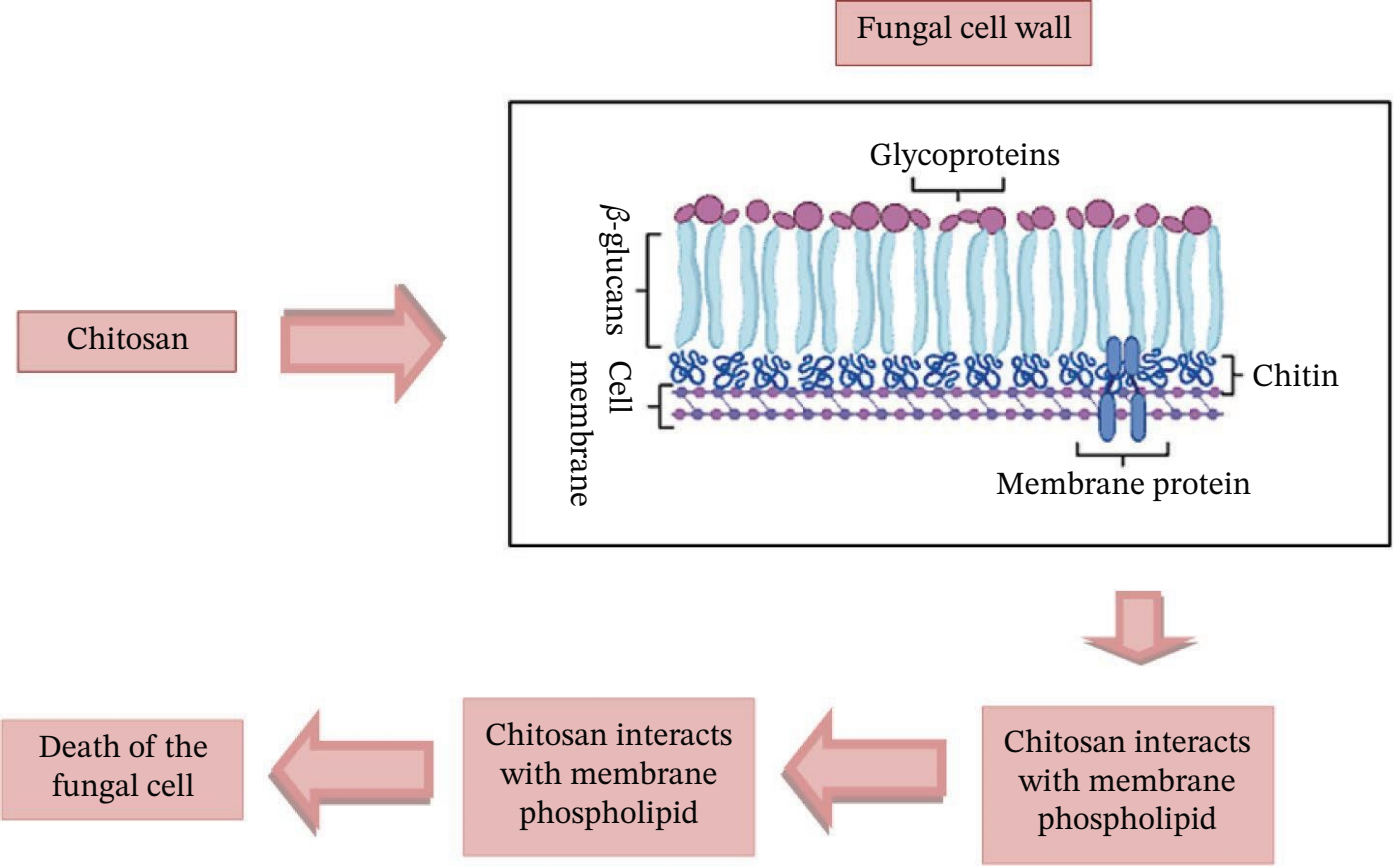
Proposed antifungal action of chitosan. The illustration highlights chitosan′s fungistatic properties, including the suppression of spore germination, induction of morphological deformities in hyphae, and direct interference with fungal cell wall integrity.

Certain molds and yeasts, including *Fusarium oxysporum*, *Botrytis cinerea*, *Rhizoctonia solani*, *Candida lambica*, and *Phomopsis asparagi*, have shown resistance to CS. Although CS′s inhibitory effects on these microorganisms, such as spore germination and growth limitation, tend to be more fungistatic than fungicidal, its mechanism of action primarily targets the cell wall structure to impede fungal growth (Figure 7). Interestingly, CS demonstrates a faster action against fungi compared to bacteria. However, the effectiveness of CS against fungi, as measured by its minimum inhibitory concentrations (MICs), can vary significantly based on factors like Mw, DD, solvent pH, and the specific type of fungus being targeted [83]. Some CS derivatives, such as quaternary CS derivatives and those containing quaternary phosphonium groups, have exhibited enhanced antifungal properties, suggesting their potential as fungicidal agents. For instance, Li et al. [84] revealed that certain quaternary CS derivatives achieved a remarkable 100% inhibitory rate against *Cladosporium cucumerinum* and *Monilinia fructicola*, underscoring the promising antifungal capabilities of these derivatives. Similarly, Wang et al. [71] discussed the antifungal potential of CS derivatives containing quaternary phosphonium groups, further highlighting the diversity of CS‐based antifungal agents.

Khare et al. [85] conducted a study revealing that the application of CS to chicken noodles effectively prevents microbial growth during storage, thereby extending their shelf life. In a separate study, Mellegård et al. [74] observed that CS with lower acetylation levels exhibited stronger inhibition of *B. cereus* growth compared to CS with higher acetylation levels. Interestingly, they also found that medium to high Mw CS demonstrated superior A_M_A_C_ against *B. cereus* compared to LMCS. However, this trend did not hold for CS with higher acetylation levels. Moreover, their research showed no significant variations in CS susceptibility among different strains of *B. cereus,* indicating consistent effectiveness across various strains.

Lafarga et al. [86] unveiled that infusing bread with a 0.6% CS powder effectively curbed the proliferation of *B. cereus* over 3 days at 30°C. Similarly, Bostan and Mahan [87] revealed that CS, in varying concentrations, markedly diminished the cell counts of diverse bacteria, including mesophilic and psychrotrophic bacteria, lactic acid bacteria, yeasts, and molds, throughout a 60‐day storage at 4°C in meat sausage. Further research by Aquino et al. [88] exhibited consistent inhibition zones against both Gram‐positive/negative bacteria in samples with differing CS concentrations. Meanwhile, Wang et al. [89] explored the implications of incorporating honeysuckle flower extract (HFE) on the physical and mechanical attributes of CS film, noting significant inhibition zones against *E. coli* in CS films lacking HFE. Despite the innate A_M_A_C_ of CS, Pranoto et al. [90] emphasized that its impact was evident without the dispersion of active substances, with Coma et al. [91] underscoring that CS′s inhibitory effects are confined to organisms in direct contact with its active sites. Also, Du et al. [92] underscored the promising inhibitory potential of both crude and water‐soluble CS against various bacterial strains, with water‐soluble CS demonstrating notably robust A_M_A_C_ compared to crude CS against specific bacteria strains.

In summary, A_M_A_C_ of CS is intricately linked to the pH of its environment, with lower pH generally enhancing its efficacy. Understanding the underlying mechanisms and factors influencing A_M_A_C_ of CS is crucial for harnessing its potential as a natural preservative in the food industry and beyond. Also, CS′s effectiveness against Gram‐negative bacteria appears to stem from its complex electrostatic interactions with the bacterial surface. Although shedding light on A_M_A_C_ of CS, these findings highlight its potential as a natural agent for combating various bacterial pathogens [93].

### 3.5. A_X_A_C_ of CS

The surge in global mortality rates attributable to diseases like cancer and cardiovascular disorders has been increasingly linked to oxidative stress, prompting a growing awareness of the importance of dietary antioxidants in combating these health threats [94]. Although synthetic antioxidants have historically been employed to counter LIP_O_ in food products, concerns about their safety have led to a shift toward natural alternatives [95]. This shift in consumer preferences has spotlighted CS and its derivatives as promising candidates due to their demonstrated A_X_A_C_, which encompasses scavenging various oxygen radicals like hydroxyl, superoxide, alkyl, and persistent DPPH radicals in lab settings [96]. As an example, Sun et al. [97] further elaborate on the A_X_A_C_ of CS derivatives, attributing their efficacy to donate hydrogen and impede the oxidative process. Such findings have fueled interest in exploring the applications of CS as a natural antioxidant across diverse fields, ranging from food preservation to biomedical research, underscoring its versatility and potential impact on human health.

A deeper examination of A_X_A_C_ of CS reveals its reliance on distinctive attributes such as DD and Mw. According to Park et al. [96], LMCS with higher DD demonstrate superior efficacy compared to those with larger Mw and lower DD. This superiority is attributed to the heightened ion chelating capacity of highly deacetylated CS. Conversely, Qin et al. [20] argue that HMCS forms a densely packed structure with robust H‐bonds, limiting the exposure of amino and hydroxyl groups and consequently diminishing radical scavenging. Supporting this notion, Sun et al. [98] observed an inverse correlation between CS′s Mw and its A_X_A_C_, with LMCS exhibiting heightened efficacy. Additionally, Yin et al. [99] found that LMCS can scavenge > 80% of superoxide radicals at 0.5 mg/mL. In their investigation of CS with varying Mw (30, 90, and 120 kDa), Kim and Thomas [54] note that all samples display A_X_A_C_, with the 30‐kDa variant showcasing the highest activity in mitigating LIP_O_ in Salmon.

Furthermore, Jeon et al. [55] highlight the superior radical scavenging of highly deacetylated (90%) CS against DPPH, hydroxyl, superoxide, and carbon‐centered radicals. Although the precise mechanism remains elusive, it is postulated that the interaction of amino and hydroxyl groups in CS with unstable free radicals leads to the formation of stable macromolecule radicals. Moreover, A_X_A_C_ of CS can be augmented through synergistic combinations with other natural substances like glucose, without compromising its A_M_A_C_ against various strains including E. *coli, S. aureus, Bacillus subtilis*, and *Pseudomonas*. Additionally, CS′s inherent property of poor oxygen permeability serves to retard LIP_O_, positioning it as a promising ingredient for prolonging shelf life of diverse food products [100].

Research on A_X_A_C_ of CS reveals a nuanced interplay of factors, particularly Mw and DD. Schreiber et al. [100] underscore the modest DPPH scavenging capacity of native CS (Mw: 307 kDa, DD: 80%), contrasting with the remarkable enhancement observed upon gallic acid grafting. Limited DPPH suppression by CS variants (Mw: 121 kDa, DD: 80%) is possibly linked to intermolecular H‐bonds in HMCS. Conversely, Li and Xia [101] showcase heightened A_X_A_C_ with epigallocatechin‐functionalized CS. Woranuch and Yoksan [102] uncover moderate DPPH scavenging in CS (Mw: 200 kDa, DD: 90%), augmented upon ferulic acid conjugation. Additionally, Pasanphan and Chirachanchai [103] highlight diverse A_X_A_C_ across CS conjugates, signaling avenues for enhancing CS′s A_X_A_C_. Furthermore, Shen and Kamdem [104] investigate CS films integrated with EG essential oil, revealing a concentration‐dependent surge in scavenging prowess. These insights underscore CS′s versatility in antioxidant applications, promising multifaceted benefits across various domains.

Studies investigating A_X_A_C_ of CS have revealed intriguing findings regarding its effectiveness under various conditions. For instance, MA et al. [105] explored different CS preparation techniques and observed varying scavenging capacities, ranging from 41.86% to 59.16%, depending on concentration and method. Similarly, Chen et al. [106] found that A_X_A_C_ of silver/CS composites increased from 20.4% to 78.6% with escalating concentrations. In contrast, Kanatt et al. [107] reported negligible A_X_A_C_ for CS alone, a result corroborated by Schreiber et al. [100] who noted minimal primary A_X_A_C_ in non‐grafted CS. On the other hand, Mahae et al. [108] highlighted the superior A_X_A_C_ of CS‐sugar complexes compared with CS alone. Additionally, Rambabu et al. [109] demonstrated significant DPPH scavenging in CS film formulations, further indicating the potential of CS‐based materials as antioxidants. Studies investigating A_X_A_C_ of CS yield mixed results. Some suggest that CS only shows a moderate A_X_A_C_, whereas others find no effect at all. However, one consistent finding is that when phenolics are grafted onto the CS structure, their A_X_A_C_ is enhanced. This means that even if the original CS does not demonstrate strong A_X_A_C_, modifying it through grafting can lead to improved efficacy.

### 3.6. CS as a Carrier for Bioactive Ingredients

Beyond its intrinsic preservative qualities, CS is increasingly utilized as an encapsulation matrix for other bioactive compounds. Its ability to form nano/microparticles via ionic gelation (e.g., with tripolyphosphate) allows it to carry essential oils, vitamins, and phenolic compounds. This “carrier” function protects sensitive bioactives from degradation during processing and allows for controlled release, thereby enhancing the overall antioxidant and antimicrobial profile of the food product.

## 4. Effect of CS Fortification on Different Food Quality Parameters

### 4.1. Proximate Composition

The proximate composition of dry pasta is already mentioned. However, the addition of tilapia flour (TL) or surimi decreased MC and carbohydrate content (*p* < 0.05) whereas increased lipid, protein, and ash (*p* < 0.05) potentially due to the tilapia surimi powder compositions. TL has a low carbohydrate content (< 1.5%) and high protein (> 45%), lipid (> 25%) and ash (> 3%) [110, 111], but wheat flour has a high carbohydrate content (> 75%) and low protein (< 11%), lipid (≤ 1.5%) and ash (≤ 0.38%) [112]. Furthermore, lower MC induced by TL might be related to a stronger protein–polysaccharide interaction when compared to wheat equivalents, which leads to protein denaturation under heating conditions, facilitating intermolecular network formation [5]. Electrostatic interactions between polysaccharides and proteins promote water entrapment, resulting in a more homogeneous network with less free water [5, 113], which is linked to a lower MC in foods high in proteins and polysaccharides. However, despite the findings of Devi et al. [114] and Anbudhasan [115] that fish‐enriched pasta has a higher MC, GOES et al. [116] found no change in MC between pasta formulations with and without fish protein concentrate (FPC). The variance in MC of pasta enriched with fish byproducts reported in the literature can be related to formulations and processing methods [115].

The MC of Acetes‐based functional snacks supplemented with CS increased significantly (*p* < 0.05) in the proximate/centesimal composition [9]. MC of the control sample was 4.12%, whereas MC of CS‐fortified samples was 4.23%, 5.28%, and 5.40%, respectively, which increased at higher CS levels, attributed to CS′s water holding capacity (WHC). CS coating provides a protective barrier for moisture transmission through food surfaces, according to No et al. [117]. But Klinmalai et al. [118] reported that CS had no significant difference in MC of flat rice noodles during storage at 30°C for 5 days, same in the case of control and bread containing 0.5% CS as it was a very low CS. A significant difference (*p* < 0.05) was observed in moisture loss when 1%, 1.5%, and 2% CS were added to the dough [119]. At higher CS levels, the crude protein content of CS‐enriched functional snacks also increased considerably (*p* < 0.05) [9]. This might be attributed to CS′s nitrogen content (about 7%), which can be added to the overall nitrogen content [120]. Other characteristics (e.g., crude fat and mineral content) did not change substantially (*p* < 0.05) when CS was added. Carbohydrate content was observed to decrease at higher CS levels [9].

### 4.2. Changes in pH

One of the chemical elements that affect the quality, safety, and freshness of fish is pH. Previous research has found that the pH of fish decreases and then gradually increases after storage [121]. CS can assist fish and other seafood items to keep their physicochemical features. The use of CS‐based coatings on seafood dramatically minimizes the accumulation of alkaline compounds, suggesting that CS helped to limit the generation of alkaline compounds by preventing microbial deterioration [120]. The use of CS‐based coatings in conjunction with other preservatives on seafood has a similar effect on pH stability. Lower pH inhibits microbiological development, extending shelf life of the product. Chattopadhyay et al. [122] showed that the CS‐included sausage samples had lower pH both before and after cooking compared with the control. The sausages with 0.375% and 0.5% CS had the lowest pH. Acetic acid used to dissolve CS results in lower pH for sausages using CS batter. [58] found that cod fillets treated with CS dissolved in acetic acid had low pH. Xavier et al. [123] found that fish sticks enrobed with varying amounts of CS had a similar pH (6.30–6.34). Furthermore, pH before cooking for all groups was considerably lower than the pH after cooking. Rajalekshmi and Mathew [124] reported that pH of all surimi samples with CS showed a slight decrease during frozen storage up to 4 months and increased slightly thereafter. During storage at 30°C for 5 days, pH of rice flour noodles containing CS remained stable [118]. CS‐incorporated hamburgers presented higher pH than the remaining hamburgers for zero days and remained high along the storage time, varying from 6.6 to 6.65. This fact could be attributed to the high pH of CS added to it [125].

### 4.3. PV

PV is a valuable and dependable tool for estimating the rate of auto‐oxidation in the early stages [126]. PV up to 30 mEq O2/kg of fat is regarded as ideal, with no disagreeable aftertaste or odor [127]. Wang et al. [128] found that PV decreased considerably (*p* < 0.05) when surimi (10%–50%) was added. Mohamed et al. [129] used FPC in biscuits and discovered that PV grew progressively in all samples till the end of storage. Jeyasanta et al. [130] published similar results, demonstrating that PV remained below the permissible range of 10–20 mEq/kg of fat during storage. The reduction in PV is primarily linked to the metal‐chelating capability of CS. By binding transition metal ions (such as Fe^2+^ and Cu2^+^), CS prevents them from catalyzing the initiation step of lipid peroxidation. In comparison to the control noodles, Ahlawat et al. [131] created extruded (noodles and macaroni) products including high protein okara (soya bean milk byproduct) which revealed slightly higher PV during the storage. In CS (1% w/w) added fresh pork sausages, Soultos et al. [132] observed the retardation of LIPO.

### 4.4. TVBN

TVBN, which is mostly constituted of ammonia and primary, secondary, and tertiary amines, is the outcome of microbial activity causing the breakdown of proteins and nonprotein nitrogenous substances [133]. Because its rise is associated with deterioration by endogenous enzyme activity and bacterial development, TVBN is well documented as an excellent measure of the quality of fresh or frozen fish and fish products. The use of CS on seafood has been demonstrated to delay TVBN levels because of microbial growth suppression, which is thought to be the source of oxidative deamination of nonprotein nitrogen molecules. Several studies reported comparable outcomes when using CS‐based coatings on other types of seafood, either alone or combined with other preservation agents [121, 133, 134]. The acceptable level of TVBN, according to Connell (1980), is 20 mg/100 g, but the permitted limit of TVBN, according to Ghaly et al. [135] is 35 mg/100 g in food. Surimi produced from Japanese threadfin bream flesh had an initial value of 6.10 mg volatile nitrogen and 0.88 mg trimethylamine nitrogen, according to Karthikeyan et al. [136]. Also, Fawzya et al. [137] found that TVBN values in bread fortified with surimi flour enhanced during storage. Ojagh et al. [138] claimed all TVBN readings were below the acceptable limit in CS‐coated samples throughout the refrigerated storage (4°C) of 16 days. [55] found that during 12‐day storage, TVBN production was reduced by 33%–50% and 26%–51%, respectively, using cod and herring fillets and different soluble CS coatings. Tsai et al. [56] also discovered that pretreatment of fish fillets (*O. nerka*) with a 1% CS solution for 3 h slowed the growth in TVBN. López‐Caballero et al. [58] showed that the protective CS–gelatin coating significantly reduced TVBN readings and thereby slowed rotting.

### 4.5. TBARS

TBA is a commonly used indicator for determining the degree of LIPO or oxidative stress.[55] According to O′Connell and Garner [139], the safe amount of TBA is 1–2 mg of malonaldehyde equivalents/kg sample. [140] reported that TBARS levels differed substantially (*p* < 0.05) between treated and control noodles, and both increased after storage. Over 30‐day storage, the total mean values of TBARS rose considerably (*p* < 0.05). Increased LIP_O_ and the formation of volatile metabolites in the presence of oxygen might explain the rise in TBARS readings as storage time progressed. Verma et al. [140] observed similar findings with chicken meat noodles. Throughout the storage, Jeon et al. [55] detected lower TBARS in CS‐coated herring and cod samples than in uncoated ones. Ojagh et al. [138] observed that TBA values of CS‐coated samples increased significantly (*p* < 0.05) with storage time. The CS‐added hamburgers presented similar (*p* > 0.05) TBARS values to Control during the frozen storage [141]. Evaluating TBARS value of Acetes‐based extrudates revealed that CS‐fortified samples have much less LIP_O_ than the control.[9] TBARS decreased considerably as CS level rose. The protective impact of CS as a hydrogen donor and oxygen barrier on LIP_O_ may be responsible for the lower TBARS result [142, 143].

### 4.6. TPC

Viable cell densities of amaranth homemade fresh pasta were observed at 4°C for roughly 2 months by [144] The findings imply that modified atmosphere packaging and CS have a combined impact in preventing the microbial spoilage of pasta during storage. Incorporation of CS (10 kDa) in surimi at 2% (w/w) also reduced the aerobic plate counts in surimi gel made from African catfish (*Clarias gariepinus*) by 1 log (CFU per g) after storage at 4°C for 12 days [145]. [66] observed that microbial counts in CS‐treated white rice cakes were below the critical level even after 76 days of storage. Park and Chong [146] also showed that adding CS to rice cakes extends shelf life. At 0.05%, 0.1%, 0.3%, and 0.5%, water‐soluble CS dissolved in water was added to rice cake. TPC decreased at higher CS concentrations after 4 weeks of storage at 5°C. TPC of rice cakes containing 0.3% and 0.5% CS were 2‐log cycles lower after 4 weeks of storage than the control (8.2 × 10^4^ CFU/g). Kok and Park [147] claimed that throughout the 21‐day storage, CS (1%) dissolved in acetic acid was most effective in inhibiting the growth of microorganisms identified in fish balls.

### 4.7. Cooking Qualities

The cooking quality of pasta is assessed by optimal cooking time (OCT), cooking loss (CL), water absorption index (WAI), and swelling index (SI). The quality and protein content and their interactions are considered one of the important factors in carbohydrate–protein networks of pasta [148–150]. Also, cooking quality is related to the ability of spaghetti to maintain textural properties during cooking [144]. Mao and Wu [151] found that adding 1% CS to a restructured grass carp surimi gel improves WHC, owing to the higher CS‐water interactions during surimi gelling. Zhang et al. [152] observed that when CS was added to PHBN (purple highland barley noodles), it would embed in the starch‐protein network, forming a relatively stable 3D structure of protein‐CS‐starch, and proton exchange would occur among protein, CS, starch, and water, which would be more powerful to restrict water molecule movement and increase shelf life.

WAI measures how much water is absorbed while submerged in water. Because CS is a polysaccharide of animal origin with a structure comparable to plant cellulose and a high number of insoluble fibers compared to hydrocolloids of vegetable origin, the CS added (M/OACHIT) noodles revealed a high‐WAI (125%) [153]. OCT is the time necessary to obtain complete gelatinization of starch [154]. Furthermore, Padalino et al. [153] observed an OCT of 9.5 min for the CS‐fortified (M/OACHIT) noodles. Kaur et al. [155] noticed OCT was less for pasta that contained cereal bran compared to durum wheat semolina pasta. They found that OCT was 5:38 min. For control pasta, which was reduced to 5:24, 5:22, 5:17, and 5:22 min. For wheat, rice, barley, and oat bran enriched pasta at the 25% supplementation. The water absorbed by starch and proteins during cooking is measured by SI of pasta, which is used for starch gelatinization and protein hydration. The CS‐added sample (M/OA‐CHIT) recorded SI (1.6) very comparable with the control [153]. The packing, storage, and transportation elements are all tied to bulk density. Encapsulated squalene powder has a bulk density = 0.582 g/L, which is fairly high. Because they take up less space, products with a greater bulk density may be kept in bigger quantities in tiny containers. Another advantage is that high bulk‐density goods have less blocked air, resulting in reduced oxidation of the end product.

CL is defined as the loss of water and fat during the cooking process. The ability of the system to bind water and fat following protein denaturation and aggregation is determined by CL. Lower CL is typically indicative of high‐quality food [156]. CL in the control sample was 1.19%, whereas it ranged from 0.66% to 1.71% in sausages combined with a hydrogel containing CS [122]. In general, CS‐treated samples had a considerably lower CL than the control, showing that CS engaged in WHC, leading to lower water losses and, as a result, weight losses during thermal processing. Padalino et al. [153] found CL = 6.1*%* in the CS added (M/OACHIT) noodles.

### 4.8. Color Properties

Generally, consumers like pasta with a bright color. Color of pasta without additives depends on the properties of flour used (e.g., carotenoids and the composition of proteins) [157]. Therefore, maintaining color within consumer acceptance levels is essential. [152] reported that the whiteness of PHBN was unaffected by CS (0.25%–2%, w/w). Klinmalai et al. [118] discovered that adding CS to rice noodles reduces their whiteness owing to the color of CS, which changes from yellow to deeper yellow after high‐temperature treatment due to the Maillard reaction, also discovered by Yang et al. [158]. They hypothesized that the amino group of CS interacted chemically with reducing sugars in PHBN at 93°C, resulting in Maillard reaction products. This impact of CS, however, was not seen on PHBN. The rich violet hue of PHBN was due to the abundance of anthocyanins in the compound [152]. They hypothesized that the effects of CS were insufficient to cause a significant change in the hue of PHBN. Hautrive et al. [141] found that during storage, the yellow hue (h_ab_∗) increased in all samples including CS incorporated one. The slow oxidation of myoglobin and buildup of metamyoglobin caused the steady decline in *b* ∗. Rajalekshmi et al. [124] reported that all samples with CS or cryoprotectants or both did not affect the color. From the color analysis results, it is evident that the sausage color was enhanced due to addition of CS [122]. These color characteristics are highly demanded by consumers as described by [159].

### 4.9. Texture

Texture profile analysis (TPA) is one of the objective methods of sensory analysis. TPA was implemented by [160] who defined the textural parameters. A typical two‐cycle TPA is used to determine many texture parameters like hardness, cohesiveness, springiness, chewiness, gumminess, adhesiveness, and resilience. [152] displayed that CS improved the textural parameters of PHBN, including hardness, gumminess, and chewiness, presumably due to covalent and noncovalent interactions of CS with protein and starch, resulting in the creation of strong gel networks. Because of the viscosity caused by CS, the adhesiveness of PHBN was greatly enhanced. CS did not influence the cohesion or springiness. The hardness, gumminess, and chewiness of PHBN were considerably improved when CS content was raised from 0.25% to 2% (w/w), showing that CS appeared to boost the gel strength of PHBN in a dose‐dependent manner. Slower changes in the textural parameters of the noodles with CS in rice flour gel might have been attributable to the lesser retrogradation of starch due to interaction of CS in the starch network during storage at 30°C for 5 days [118].

According to Hajidoun and Jafarpour [161], adding CS to carp surimi gels improves TPA parameters (e.g., hardness, adhesiveness, and elasticity) because CS prevents the degradation of the myosin‐heavy chain by facilitating cross‐linking, which stabilizes the protein gel network, allowing for the formation of cross‐linked myosin heavy chain components. CS appears to operate in food by building stronger protein gel networks and also by generating H‐bonds and electrostatic interactions. Li and Xia [101] discovered that the interaction between CS and salt‐soluble meat proteins leads to the enhanced texture of salt‐soluble meat proteins. Under dry heat treatment, the amino groups of CS can cross‐link with the hydroxyl groups of starch, causing the starch granules to expand, according to Diao et al. [162]. Hardness, springiness, and cohesiveness were all greater in the CS‐included hamburger. This might be attributed to the hamburger′s poorer moisture retention and increased fat retention after cooking. Although the CS‐containing sample was springier than the other hamburgers, it was also harder, which might be due to lower MC in the cooked hamburger and reduced WHC after cooking [141]. The chemical composition of the polysaccharide components, as well as other parameters such as porosity, particle size, shape, ionic force, pH, temperature, type of ions in solution, and tension between fibers, affect diet fiber hydration qualities [163].

### 4.10. Sensory Attributes

Based on characteristics (e.g., appearance, aroma, flavor, firmness, and overall acceptability), the influence of CS on sensory attributes is assessed [164]. Kumar et al. [9] compared Acetes‐based extrudates fortified with different CS levels; the overall acceptance of samples fortified with 1% CS was significantly higher (*p* < 0.05). All the sausages, with or without CS, received high marks for sensory scores in another study [122]. Similar findings were made by Lin and Chao [51] and do Amaral et al. [165] who found that CS had neither detrimental nor beneficial influence on the sensory qualities of freshly produced sausages. The analysis of CS‐coated cooked rainbow trout fish fillet was imperceptible and did not produce undesirable sensory properties. On the other hand, CS coating significantly (*p* < 0.05) enhanced the color and overall acceptability of the raw fish fillet in refrigerated storage by controlling LIP_O_ and microbial growth. According to Hautrive et al. [141] the hedonic averages for all the qualities under consideration ranged from 5.5 to 7.3, suggesting that consumers rated the products as “indifferent” to “I liked it a little,” with the CS‐containing group receiving lower (*p* < 0.05) ratings than the control.

The sensory properties of PHBN were significantly changed (*p* < 0.05) by CS [152]. All sensory features of PHBN with 0.25% CS did not differ from the control, indicating that CS < 0.25% was too low to significantly alter PHBN sensory attributes. The addition of CS (0.5%–1%) boosted the hardness, flavor, and overall acceptability, which might be due to the strong gel strength caused by the cross‐linking of CS, starch, and protein in PHBN [162]. The addition of 2% CS, on the other hand, dramatically reduced the hardness and viscosity of PHBN. The poor flavor and mouthfeel of PHBN were caused by the excessive stiffness and viscosity created by 2% CS, drastically lowering its taste and overall acceptability. Food taste perception is based on input from several sensory afferents, including gustatory, olfactory, and somatosensory fibers, according to Sreerekha et al. [166] Due to its odorless nature, CS did not affect the olfactory character (aroma) of PHBN, but it did considerably lower gustatory satisfaction due to the excessive stiffness and viscosity caused by 2% CS, resulting in a reduction in PHBN flavor. Ghoshal and Mehta [119] also discovered that 2% CS greatly reduces bread taste. Accordingly, [152] reported that 1% CS was the most appropriate level, which not only boosted shelf life and sensory qualities of PHBN but also lowered postprandial glycemia. The bread made of 1% CS was found to be a better option according to [119]. Table 1 presents an overview of changes in the quality of different food products containing CS.

**Table 1:** Effect of chitosan (CS) incorporation on food quality.

Product	Chitosan type/conditions	Method of application	Concentration	Key results (technofunctional and shelf life)	Ref.
Fresh pork sausages	CS (490 kDa)	Incorporation	1% (w/w)	Significant retardation of lipid oxidation (LIPO); TBARS values remained lower than control during storage.	[132]
Surimi gel (African catfish)	CS (10 kDa)	Incorporation	2% (w/w)	Reduced aerobic plate counts by 1 log CFU/g; extended shelf life by 4 days; retardation of LIPO.	[145]
Mayonnaise	CS added to formulation	Mixing	Not specified	Altered texture and flavor intensity; achieved most favorable sensory scores after 4 months of storage.	[167]
Rice cake and wet noodle	Water‐soluble CS	Dipping/coating	0.1% and 0.2%	Extended shelf life significantly (> 82 days) compared to control; maintained microbial counts below threshold.	[64]
Fish sausage	CS	Coating	1.5%	Increased elasticity and yellowness; TVBN levels remained steady for 25 days.	[58]
Rice flour noodles	CS in acetic acid	Addition	0.33% and 0.50%	Inhibited increases in hardness and gumminess; stabilized pH; reduced cohesiveness and springiness loss during 5‐day storage.	[118]
Salmon fish	CS solution	Pretreatment/coating	1%	Inhibited TVBN formation and microbial growth; extended shelf life from 5 to 9 days.	[56]
Minced herring	CS (14, 57, and 360 cP)	Mixing	50–200 ppm	Significantly reduced lipid oxidation (LIPO) dependent on viscosity and concentration.	[53]
Croaker surimi	CS as cryoprotectant	Addition	1%	Stabilized muscle protein during frozen storage; improved gel strength without compromising overall acceptability.	[168]
Purple highland barley noodles (PHBN)	CS	Incorporation	0.25%–2% (w/w)	Improved hardness, gumminess, and chewiness; 1% concentration was optimal for sensory quality; 2% negatively impacted taste.	[152]
Bread	CS (Mw: various)	Incorporation	0.1%–2% (w/w)	Reduced moisture loss; 1% CS identified as optimal functional ingredient; higher concentrations (2%) reduced taste acceptability.	[119]
Extruded snacks	CS added to corn/rice flour	Fortification	1%	Increased moisture content (4.12%–5.40%) and protein; significantly reduced TBARS (oxidation) and improved functional quality.	[9]
Fish mince emulsion sausages	CS hydrogels	Hydrogel inclusion	0.125%–0.5%	0.25% yielded best properties; reduced cooking loss (0.66%–1.71% vs. 1.19% control); enhanced color.	[122]
Meatball	Shrimp skin CS	Coating vs. mixing	1.5%	Coating extended shelf life by 4 days based on sensory analysis; effective natural preservative.	[169]
Catfish fillet	CS	Vacuum tumbling/spraying	Not specified	Reduced pH, TVBN values, and LIPO; improved microbial shelf life.	[170]
Walleye pollock surimi	CS	Addition	1%–1.5%	Improved heat‐induced gel strength in low‐quality surimi.	[171]
Kamaboko gels	CS	Addition	1% (10 mg/g)	Improved gelling qualities and overall gel strength.	[151]
Fish ball	CS dissolved in acetic acid	Addition	1%	Maintained aerobic plate and yeast counts at < 1 log CFU/g throughout 21 days of storage.	[147]

## 5. Safety and Regulatory Considerations

CS, extensively researched for its safety profile, has shown low toxicity and high tolerability even at high doses, as evidenced by Xu and Du [172]. Both acute toxicity tests and long‐term chronic exposure studies have failed to reveal significant adverse effects. Furthermore, No et al. [173] highlighted CS′s biodegradability and nontoxic nature, positioning it as a natural and sustainable food additive. Regulatory bodies, including the Food and Drug Administration (FDA), the European Food Safety Authority (EFSA), and the Joint FAO/WHO Expert Committee on Food Additives (JECFA), have all granted approvals for CS. FDA has awarded it generally recognized as safe (GRAS) status, signifying its adherence to stringent safety standards and its suitability for various food applications [29]. These regulatory endorsements solidify CS′s reputation as a safe and reliable natural preservative, aligning with consumer preferences for clean‐label ingredients while maintaining high safety standards in the food industry [174]. Moreover, recent studies have underscored CS′s nontoxic nature, affirming its safety even at higher concentrations and emphasizing CS′s biodegradability and environmentally friendly attributes, further bolstering its appeal as a natural preservative [166, 175]. These regulatory validations not only reassure consumers but also underscore CS′s pivotal role in ensuring food safety and quality while adhering to stringent regulatory standards.

## 6. Limitations and Future Challenges

Although CS demonstrates significant potential, there are limitations to its widespread industrial adoption. The cost of production for high‐purity CS remains higher than synthetic preservatives. Furthermore, the variability in natural sources leads to batch‐to‐batch inconsistency in Mw and DD, which directly alters antimicrobial efficacy. Regulatory hurdles also exist; whereas GRAS in the US, regulations vary globally regarding permitted daily intake. Finally, the astringent taste of CS at higher concentrations can negatively impact sensory acceptance, necessitating careful optimization of fortification levels.

## 7. Conclusion and Future Research

The exploration of CS′s potential as a food additive and preservative has sparked considerable interest among food scientists and technologists, leading to innovations such as the fortification of cereal‐based products with CS. However, further research is warranted to establish standardized fortification rates, determine Shelf life of fortified foods, and assess the feasibility of the fortification process. Although CS exhibits promise as a food preservative and edible coating material, variations in its physicochemical attributes from different suppliers or sources present a challenge. Ensuring uniform characteristics of CS globally requires the development of cost‐effective and reliable analytical procedures for quality control during manufacturing. Moreover, modifications to traditional CS production methods can impact its physicochemical and functional properties, necessitating further investigation into alternative manufacturing techniques. The Mw of CS plays a crucial role in its effectiveness as a food preservative, highlighting the need for research on novel manufacturing approaches (e.g., ozone technology) to tailor CS to specific applications. Additionally, efforts to mitigate the slightly bitter taste of CS through the incorporation of flavor‐masking agents like L‐arginine and AMP warrant exploration to enhance its acceptability as a food additive.

CS′s biodegradability and A_M_A_C_ make it an attractive material for biodegradable antimicrobial packaging. However, challenges such as sensitivity to humidity and poor mechanical characteristics of CS films necessitate further study to develop more robust packaging solutions. Incorporating antibacterial/antioxidant compounds into CS films or coatings may further enhance their efficacy in preserving fresh‐cut fruits and vegetables. Further research is also needed to determine shelf life of CS‐fortified or CS‐coated foods under commercial settings, paving the way for their widespread commercialization. Additionally, exploring the bioavailability of CS in cereal‐based products and its impact on nutritional quality can provide valuable insights into its potential as a functional food ingredient.

Despite its numerous advantages, CS faces challenges related to low solubility and variability in molecular structure, which affect its biological and technical performance. Investigating chemical modifications to overcome these challenges and improve polymer characteristics is crucial for expanding the utility of CS in various applications. In conclusion, Although CS holds immense promise for diverse applications in food, healthcare, agriculture, and environmental remediation, addressing current challenges and advancing research efforts will be essential for realizing its full potential in addressing contemporary challenges and improving human health and well‐being. Continued interdisciplinary research collaboration and innovation will be key drivers in unlocking the vast opportunities offered by CS in the future.

**Nomenclature**
CL: cooking loss
DA: degree of acetylation
DD: degree of deacetylation
EDTA: ethylenediaminetetraacetic acid
EFSA: the European food safety authority
FAO: food and agriculture organization
FDA: Food and Drug Administration
GRAS: generally recognized as safe
HFE: joneysuckle flower extract
JECFA: Joint FAO/WHO Expert Committee on Food Additives
MIC: minimum inhibitory concentrations
MW: molecular weight
NHCOCH_3_: acetyl amine, –OH: Hydroxyl
OCT: optimal cooking time
PHBN: purple highland barley noodles
PV: peroxide value
SI: swelling index
TBA: thiobarbituric acid
TBARS: thiobarbituric acid‐reactive substances
TPA: texture profile analysis
TPC: total plate count
TVB‐N: total volatile base‐nitrogen
WAI: water absorption index

## Author Contributions

**Asik Ikbal:** writing original draft, software. **Supratim Chowdhury:** writing original draft, methodology. **Ankures Bhattacharya:** data curation, writing – reviewing and editing. **Siddhnath Kumar:** investigations, writing – reviewing and editing. **Vijay Kumar Reddy Surasani:** writing original draft, methodology, software. **Saranya Vinayagam:** conceptualization, writing – reviewing and editing. **Selvamani Palanisamy:** writing – reviewing and editing. **Lalitha Gnanasekaran:** data curation, investigations, writing – reviewing and editing. **Sowjanya Muthyam:** formal analysis, conceptualization, writing – reviewing and editing. **Pavan Kumar Dara:** supervision, formal analysis, writing – reviewing and editing. **Hitesh Chopra:** investigation, writing – reviewing and editing. **Tabarak Malik:** visualization, formal analysis, writing – reviewing and editing. We confirm that all authors agree to be accountable for the research presented.

## Funding

No funding was received for this manuscript.

## Conflicts of Interest

The authors declare no conflict of interest.

## Data Availability

The data will be made available upon request.
